# Regulation of MYB mediated cisplatin resistance of ovarian cancer cells involves miR-21-wnt signaling axis

**DOI:** 10.1038/s41598-020-63396-8

**Published:** 2020-04-23

**Authors:** Xue-yan Zhang, Yun-feng Li, He Ma, Yun-he Gao

**Affiliations:** 1 School of Nursing, Jilin University, Changchun Jilin, 130021 China; 2 Department of Radiotherapy, Second Hospital of Jilin University, Changchun 130022 Jilin, China; 3 Department of Anesthesiology, Second Hospital of Jilin University, Changchun 130022 Jilin, China; 4 Department of Pathology, Second Hospital of Jilin University, Changchun 130022 Jilin, China

**Keywords:** Ovarian cancer, Ovarian cancer

## Abstract

c-MYB has been reported to be elevated in few cancers, including in ovarian cancer. It influences resistance to cisplatin but the details are not very well understood. The objective of this study was to further evaluate role of c-MYB in ovarian cancer’s cisplatin resistance. To elucidate the underlying mechanism of cisplatin resistance in ovarian cancer, we focused on the epigenetic regulation by miRNAs. Two cell lines, ES2 and OVCAR3, were used as the model systems. C-MYB expression was either up-regulated or silenced and the resulting effect on cisplatin resistance evaluated, along with the mechanistic role of miR-21, through transfections with pre/anti-miRNAs. An *in vivo* cisplatin resistance model was also employed to verify findings. High c-MYB correlated with increased miR-21. High c-MYB also resulted in induction of EMT and increased resistance against cisplatin which was attenuated by anti-miR-200s. c-MYB decreased β-catenin phosphorylation and thus activated wnt signaling. Silencing of c-MYB resulted in reduced miR-21 levels, reduced EMT, reduced cisplatin IC-50s and increased β-catenin phosphorylation. In an *in vivo* mice model of cisplatin resistance, c-MYB overexpressing ES2 xenografts were more aggressive than their control counterparts. These c-MYB overexpressing ES xenografts were significantly more resistant to cisplatin but could be sensitized to cisplatin by anti-miR-21. Our results provide a novel mechanism of cisplatin resistance by c-MYB which involves an essential role of miR-21.

## Introduction

Among all the gynecological cancers, ovarian cancer is considered to be the most lethal^1^. Further, for the treatment of ovarian cancers, cisplatin is among the most trusted therapy with measurable clinical response^2,3^. The phenomenon of developing resistance against therapy, particularly against cisplatin-based therapy, in ovarian cancer patients has been well documented^4,5^. Researchers have focused on a number of causative mechanisms and this has led to many laboratories discussing the possible role of microRNAs (miRNAs) in cisplatin resistance of ovarian cancer^6–8^.

A protooncogene c-MYB was recently showed to contribute to cisplatin resistance in ovarian cancer^9^ and these reported results were the first ever detailing the c-MYBs contribution to proliferation, invasion and development of cisplatin resistance in ovarian cancer cells. One of the key findings of this work was that c-MYB expression is relatively higher in ovarian cancer patients, as compared to normal controls, thus clearly suggesting a role of c-MYB in ovarian cancer pathogenesis. Moreover, the expression of c-MYB correlated with higher grade ovarian cancer which suggested a direct relationship between c-MYB expression and aggressive ovarian cancer. The oncogenic role of c-MYB is not limited to ovarian cancer and has been reported in hematological malignancies as well as several solid tumors^9–13^, Despite such wealth of information regarding the oncogenic potential of c-MYB, its role in cisplatin resistance was not known prior to this report^9^. The study reported a connection between c-MYB expression and acquired cisplatin resistance, but there is still no clear understanding regarding how c-MYB can influence cisplatin resistance. It was because of this gap in our understanding that we decided to design this current study to further dissect the cisplatin resistance of ovarian cancer cells. We used ES2 and OVCAR3 as our model systems wherein we performed c-MYB transfections as well as silencing, as appropriate. Moreover, we started with a screening of miRNAs that were altered upon c-MYB transfections. Once we identified miR-21 as a candidate miRNA, we evaluated mechanism by focusing on wnt signaling pathway. Finally, we employed an *in vivo* model to further corroborate our findings.

## Materials and Methods

### Cell Lines and other materials

We purchased ES2 and OVCAR3 ovarian cancer cell lines from ATCC. OVCAR3 cell line was cultured in RPMI medium while ES2 cell line was cultured in McCoy’s 5a medium with 10% Fetal Bovine Serum. Cell lines were cultured in 5% CO_2_ humidified incubator with the temperature set to 37 °C.

### c-MYB and miR-21 transfections

c-myb cloned in pCMV6-XL5 was purchased from Origene and transfected using TurboFectin transfection reagent while siRNA against c-myb was purchased from SCBT (China). Pre-miR-21 and anti-miR-21 reagents were purchased from ThermoFisher (China) and transfected using Dharmafect reagent (Dharmacon, China).

### BrdU cell proliferation assay

We performed BrdU (5-bromo-2′-deoxyuridine) cell proliferation assay using BrdU proliferation kit (Cell Signaling). It detects BrdU that gets incorporated into the cellular DNA during cell proliferation, using an anti-BrdU antibody. The protocol provide by vendor was followed, using 3500 cells seeded in individual wells of 96-well plates with labeling medium that contained BrdU. After requisite incubation of 72 hours, labeling medium was removed and 100 μl of fixing/denaturation solution was added for half hour. Then 1X detection antibody was added for 1 hour. Plate was washed 3 times with supplied wash buffer before addition of anti-mouse IgG, Horseradish Peroxidase-linked antibody to recognize the bound detection antibody. 100 μl Horseradish Peroxidase substrate TMB (3,3′,5,5′-Tetramethylbenzidine) was added to develop color which was read at 450 nM on a Shimadzu reader (Japan).

### RNA Preparation and qRT-PCR

We used Trizol reagent to isolate RNA, by following the exact instructions provided by the vendor. The qRT-PCR reactions were performed on an ABI 7500 RT-PCR system (Applied Biosystems). We used primers and detection reagents purchased from Qiagen (China) to detect miR-21. Only RNAse-free water was used throughout the assays.

### ELISA for β-catenin

We used ELISA (Enzyme-linked immunosorbent assay) to detect p- β-catenin (TGR Biosciences, Australia), as per the instructions provided with the product. Control cells or those transfected with c-MYB in the presence or absence of pre/anti-miR-21s, were seeded overnight in a 96 well plate (5000 cells/well) in complete medium containing 10% FBS. The next day they were lysed as instructed and 50 μL of lysate transferred to 3 replicate wells of ELISA*ONE* assay plate. Antibody mix specific for phospho-β-catenin was then added to the wells and the plates incubated for 1 hour at room temperature with shaking. Then substrate mix was added, after washing, and the plates covered with aluminum foil and incubated for 10 minutes with shaking. The absorbance at 450 nM was determined using a Shimadzu plate reader (Tokyo, Japan).

### *In vivo* study

The *in vivo* experiments involving mice were performed only upon approval by the Animal Welfare Committee of Jilin University (protocol # 18-02312), and all methods were performed in accordance with the relevant guidelines and regulations. We performed these experiments using female athymic nude mice (Vital River Laboratory Animal Technology Co. Ltd., Beijing, China). Mice were maintained under specific pathogen-free conditions with free access to drinking water and housed in a restricted access room under a 12 hour light/ 12 hour dark cycle with controlled temperature environment. They were inoculated subcutaneously in the flank with 0.1 ml of cell suspension containing 2.5×10^6^ ES2 ovarian cancer cells. When tumor were visible, they were measured for size using calipers, and volume was calculated using the formula: volume = length × width^2^/2. After approximately 7 days, some mice received a ‘pretreatment’ dose (0.75 mg/kg) of cisplatin. All animals received a higher dose (3.0 mg/kg) a week later and the tumor volume measured for following few weeks, as indicated.

## Results

### c-MYB and cisplatin resistance

It has been reported previously that c-MYB greatly influences cisplatin resistance in ovarian cancer cells^9^. Our first task was to check this in our ovarian cancer model cells, ES2 and OVCAR3 cells. Both of these cells were appropriately seeded in 96-well culture plates and then treated with increasing doses of cisplatin for 72 hours so that the IC-50 values could be calculated. Table 1 shows this data that c-MYB transfection resulted in increased IC-50 values, which is indicative of induced resistance. The trend was same for both of the cell lines that we tested. On the contrary, when we silenced c-MYB, an opposite effect was observed. Now, the IC-50 values were significantly decreased (Table 1) against cisplatin. Again, the trend was exactly same for both the ovarian cancer cell lines. The consistent results in two different ovarian cancer cell lines provided us the confidence that c-MYB plays a role in determining cisplatin resistance, thus confirming the previously published work of other researchers.

**Table 1:** Effect of c-MYB and miR-21 levels on Cisplatin’s IC-50 values for ovarian cancer cells. **A**. Effect of c-MYB overexpression on Cisplatin’s IC-50 values for ovarian cancer cells, with and without miR-21 silencing. **B**. Effect of c-MYB silencing on Cisplatin’s IC-50 values for ovarian cancer cells, with and without ectopic miR-21 expression.

A	ES2		OVCAR3	
	*Control*	+*c-myb*	*Control*	+*c-myb*
	4.72 ± 0.3	16.11 ± 1.1^#^	3.21 ± 0.2	8.79 ± 0.9^#^
Anti-miR-21	4.02 ± 0.2	8.78 ± 1.0^#^	2.76 ± 0.1	4.99 ± 0.3^#^
**B**	**ES2**		**OVCAR3**	
	***Control***	**+*si-c-myb***	***Control***	**+*si-c-myb***
	4.72 ± 0.3	2.16 ± 0.1^#^	3.21 ± 0.2	2.07 ± 0.1^#^
Pre-miR-21	5.89 ± 0.4	4.27 ± 0.2^#^	5.15 ± 0.3	3.06 ± 0.2^#^

Treatments were done for 72 hours and values are μM ± SEM. #p < 0.01, compared to control.

### c-MYB effect on miRNA expression

**Figure 1:**
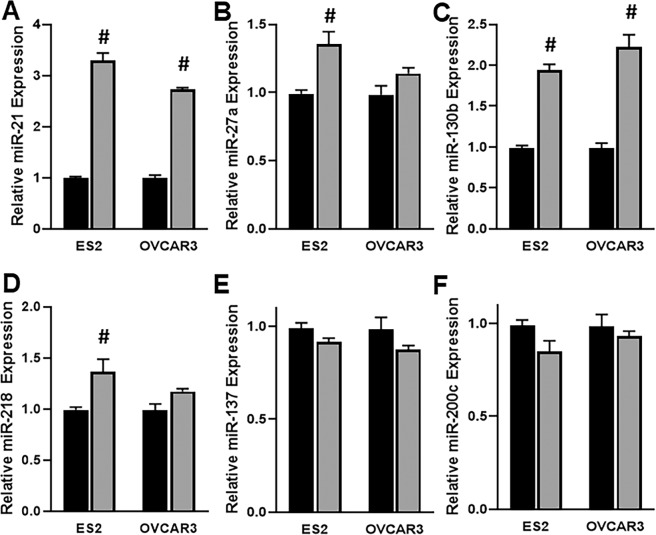
c-MYB overexpression alters the levels of several miRNAs. **(A–F)** Levels of several miRNAs, as indicated on Y-axis, were assessed, by qRT-PCR, in two ovarian cancer cell lines (ES2 and OVCAR3). Expression of miRNAs in control cells was set as 1 (black bars) and the altered expression of same miRNAs in c-MYB overexpressing cells is shown as gray bars. ^#^p < 0.01, compared to control.

In order to understand the underpinning epigenetic effect of c-MYB on cisplatin resistance, we first looked at altered miRNA expression, particularly of those miRNAs that have been reported to have an impact on cisplatin resistance. The screened miRNAs with published reports on cisplatin sensitivity that were differentially expressed upon c-MYB over-expression were miR-21^14^, miR-27a^15^, miR-130b^16^, miR-137^17^, miR-200c^18^, miR-218^19^. We found that four miRNAs, miR-21, miR-27a, miR-130b and miR-218 were elevated in c-MYB overexpressing cells, whereas two miRNAs, miR-137 and miR-200c were expressed at reduced levels in c-MYB overexpressing cells (Fig. 1). Further, the results were consistent across both the cell lines tested. We particularly observed miR-21 to be the most altered miRNA in c-MYB over-expressing cells, and, therefore, focused on this miRNA for further evaluations.

### miR-21, c-MYB and cisplatin resistance

Our miRNA screening revealed miR-21 to be the most differentially expressed miRNA when c-MYB levels were altered. Therefore, we next checked if miR-21 could reverse the effects of c-MYB on cisplatin’s IC-50 in the two ovarian cancer cells, as presented in Table 1. In first setup (Table 1), we hypothesized that since c-MYB increases IC-50 for cisplatin and also increases the levels of miR-21, the involvement of miR-21 can be established by antagonizing this miRNA in the c-MYB overexpressing cells. As seen in Table 1, when we used anti-miR-21 oligo transfections, the effect of c-MYB on cisplatin IC-50 was significantly decreased in both the cell lines. In the reciprocal setup, where we silenced c-MYB and observed reduced IC-50 values for cisplatin, we hypothesized that ectopic expression of miR-21 can abrogate these effects. This was indeed observed, as the reduced IC-50 values seen upon c-MYB silencing were almost brought back to control levels by ectopic miR-21 transfections (Table 1).

### miR-21 affect EMT

**Figure 2:**
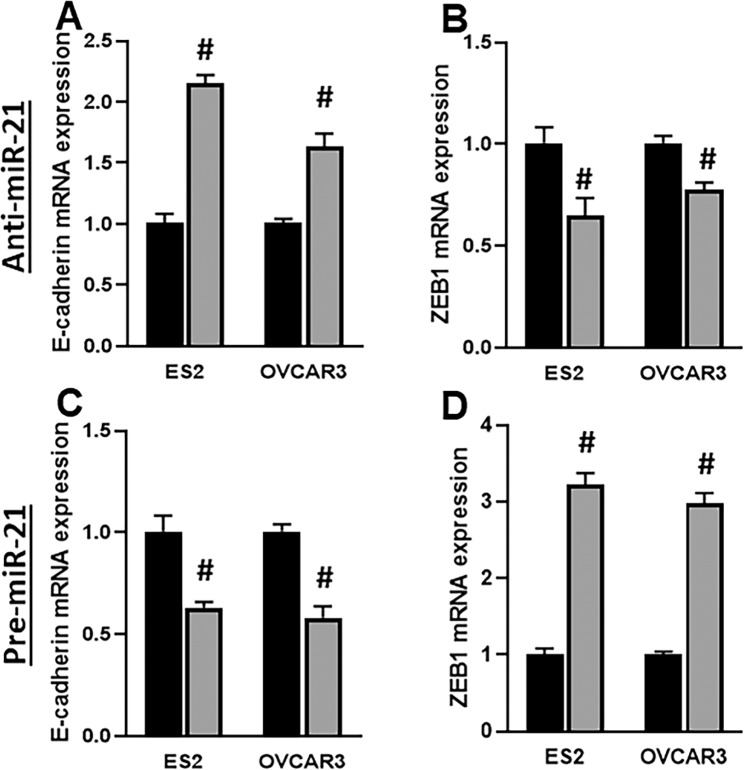
miR-21 affects EMT. **(A–D)** Levels of EMT markers, as indicated on Y-axis, were assessed, by qRT-PCR, in two ovarian cancer cell lines (ES2 and OVCAR3). Expression of genes in control cells was set as 1 (black bars) and the altered expression of same genes in presence of anti-miR-21 **(A,B)** or pre-miR-21 **(C,D)** transfections is shown as gray bars. ^#^p < 0.01, compared to control.

One of the phenomenon affected by miR-21 is that of epithelial-mesenchymal transition (EMT)^20^. To establish such effect of miR-21 in our model, we transfected either anti-miR-21 oligos or the pre-miR-21 oligos and checked for the effect on EMT induction, by quantitating the EMT markers, E-cadherin and ZEB1, qRT-PCR. Since miR-21 is elevated in cisplatin resistant cells, we first transfected cells with anti-miR-21 and found inhibition of EMT, as seen through increase in epithelial marker E-cadherin (Fig. 2A) and decrease in mesenchymal marker ZEB1 (Fig. 2B). This was further confirmed by a reciprocal experiment where transfections with pre-miR-21 induced EMT, as seen through increase in mesenchymal marker ZEB1 (Fig. 2D) and decrease in epithelial marker E-cadherin (Fig. 2C). Again, the results were consistent across both cell lines tested. So, miR-21 mediated EMT could be the cause of its cisplatin resistance inducing property.

### c-MYB, miR-21 and wnt signaling

**Figure 3:**
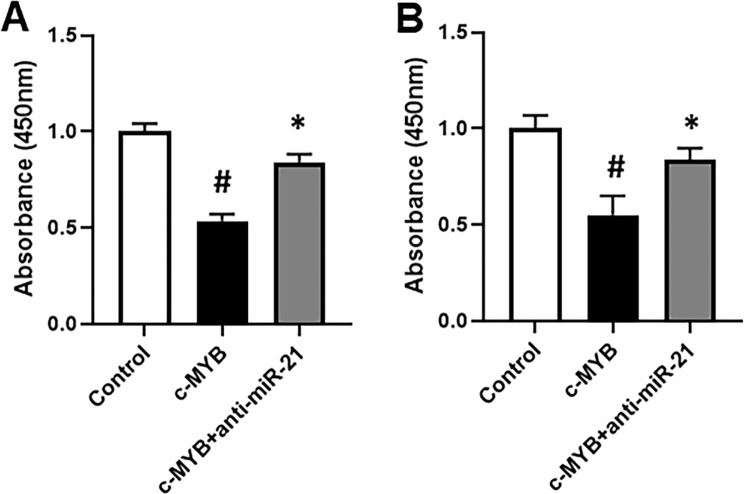
C-MYB activates Wnt signaling through miR-21. Wnt signaling was assessed through phosphorylation of β-catenin, as detected by ELISA, and endpoint measurement of absorbance at 450 nm. ES2 (**A**) and OVCAR3 (**B**) were transfected with c-MYB alone or with c-MYB and anti-miR-21, and then β-catenin was detected by ELISA. *p < 0.05 and ^#^p < 0.01, compared to control.

We observed that the miRNAs affected by c-MYB transfections, as shown above, are involved in regulating wnt signaling^14,16,17,19^, and, moreover, one of the signaling pathway affected by miR-21 is wnt signaling pathway^14^, therefore, for our mechanistic studies on cisplatin resistance by c-MYB, through miR-21, we focused on wnt signaling pathway. We evaluated β-catenin phosphorylation through ELISA. Phosphorylation of β-catenin is an indicator of diminished wnt signaling as phosphorylated β-catenin is marked for degradation. Upon c-MYB transfections, we observed decreased phosphorylation of β-catenin in ES2 cells (Fig. 3A) thus indicating activated wnt signaling. Moreover, anti-miR-21 diminished this effect of c-MYB thus proving. Similar to these results in ES2 cells, c-MYB inhibited phosphorylation of β-catenin in OVCAR3 cells as well (Fig. 3B).

### c-MYB silencing, miR-21 expression and wnt signaling

**Figure 4:**
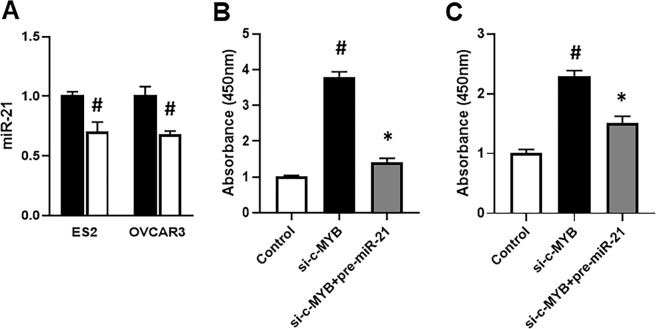
miR-21 reverses effect of c-MYB silencing on wnt signaling. **(A)** Levels of miR-21 were assessed, by qRT-PCR, in two ovarian cancer cell lines (ES2 and OVCAR3). Expression of miR-21 in control cells was set as 1 (black bars) and the altered expression of miR-21 in c-MYB silenced cells is shown as gray bars. Wnt signaling was assessed through phosphorylation of β-catenin, as detected by ELISA, and endpoint measurement of absorbance at 450 nm. ES2 (**B**) and OVCAR3 (**C**) were transfected either with siRNA against c-MYB alone or with pre-miR-21, and then β-catenin was detected by ELISA. *p < 0.05 and ^#^p < 0.01, compared to control.

Based upon our observation that c-MYB overexpression induces miR-21 and the cisplatin resistance of ovarian cancer cells, and that such condition leads to activation of wnt signaling, we further tested if silencing of c-MYB could have the reverse effects. Therefore, we silenced c-MYB, using specific siRNAs. Silencing was confirmed before conducting further experiments. First, we looked at the effect of c-MYB silencing on the expression of endogenous miR-21 and found that silencing of c-MYB in both ES2 and OVCAR3 cells caused suppression of miR-21 expression (Fig. 4A). We further evaluated wnt signaling under these experimental conditions and found that in ES2 cells (Fig. 4B), silencing of c-MYB increased the phosphorylation of β-catenin, which was almost completely attenuated by miR-21 transfections. Very similar results were observed in OVCAR3 cells as well (Fig. 4C).

### *In vivo* results

**Figure 5:**
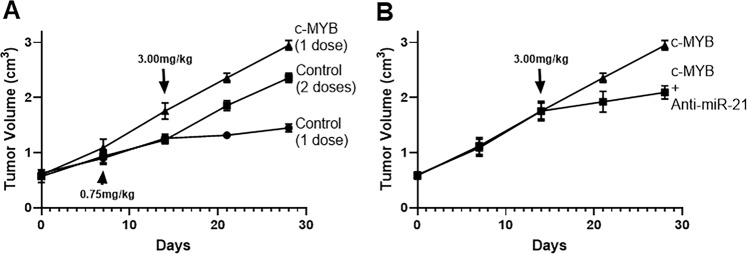
*In vivo* cisplatin resistance model. (**A**) ES2 cells xenografts were established as described in Methods and mice with control ES2 xenografts or c-MYB transfected ES2 xenografts were challenged with a single dose of 3.0 mg/kg cisplatin. A control group was administered sub-optimum 0.75 mg/kg dose to develop resistance against cisplatin. (**B**) The c-MYB ES2 xenografts were further compared to c-MYB-anti-miR-21 ES2 xenografts for tumor progression. n = 8 mice per group.

We performed an *in vivo* cisplatin resistance experiment using mice, as described before^21^. As seen in Fig. 5A, control ES2 cells receiving just one dose (high – 3.0 mg/kg) are sensitive to cisplatin while those receiving two doses (including an additional low dose (0.75 mg/kg) are resistant to high dose of cisplatin. Cells transfected with c-MYB form bigger tumors than the controls, and are resistant to cisplatin even without the administration of low doses. Further, transfections of anti-miR-21 do not seem to affect the early growth of tumor but sensitize ES2 cells to cisplatin and the single high dose slows the tumor growth (Fig. 5B).

## Discussion

Ovarian cancer is by far the most lethal cancer that affects female reproductive system. In mainland China, ovarian cancer remains the third most common cancer affecting the female genital system and the survival rates associated with this gynecological cancer are much lower than the survival rates for the other gynecological cancers such as the cervical and endometrial cancers^1^. The overall five-year survival rate of Chinese ovarian cancer is 38.9%^22^ while that of stage IV ovarian cancer in the subcontinent is 16.1%^23^. Thus, ovarian cancer is a major problem in China as well as worldwide. One of the main reasons for high lethality of ovarian cancer is the often associated acquired resistance against chemotherapies^24,25^.

For our study, we focused on miRNAs because these tiny non-coding RNAs are well known to be mechanistically involved in determination of drug resistance^26^. In particular, miR-21 is well known to be involved in resistance against therapies^27^, including resistance against cisplatin based therapy^28^. However, in spite of such activity of miR-21, its possible role in c-MYB induced cisplatin resistance of ovarian cancer has never been reported. This represents a clear gap in our understanding and underlines the novelty of current work. Through the experiments described in this current work, we have established a direct correlation between c-MYB and miR-21. c-MYB transfections elevated miR-21 levels while c-MYB silencing had an opposite effect and it led to suppression of miR-21 expression. C-MYB transfections also led to cisplatin resistance as determined by IC-50 values for cisplatin in both ES2 and OVAR3 cells. Such effect of c-MYB on induction of cisplatin resistance could be blunted by changing the expression of miR-21 as antagonizing miR-21 attenuated the effect significantly. As a further proof, silencing of c-MYB greatly reduced the cisplatin IC-50s in both ES2 and OVCAR3 cells which again verified a measurable effect of c-MYB on cisplatin sensitivity. And as a mechanism supporting role of miR-21, we were able to show, using miR-21 transfections, that miR-21 expression is almost sufficient to overcome the effect of c-MYB silencing.

We have used two different cell lines throughout our *in vitro* study which serves as confirmation and verification of our findings. Moreover, we also present an *in vivo* model of cisplatin resistance that was characterized a few years back^21^. Using this model, we show that not only the tumors in xenografts with c-MYB overexpressing cells are relatively larger but they are more resistant to cisplatin as well. This model utilizes a sub-optimal dosing of cisplatin to prime the tumors so that the tumors are refractory to a subsequent higher cisplatin dose. However, our results clearly show that xenografts with c-MYB overexpressing cells do not need such priming and are resistant to higher dose even without the ‘priming’ dose. As a further proof of an involvement of miR-21 in this cisplatin resistance *in vivo*, we show that antagonizing miR-21 does sensitize these c-MYB overexpressing tumors to cisplatin and the response almost resembles those of control xenografts.

In the current study, we initiated the investigation in an unbiased approach and screened several miRNAs for the possible role in c-MYB induced cisplatin resistance. miR-21 stood out as the most differentially expressed miRNA and was therefore chosen for further mechanistic experiments. We have reported here the six top differentially regulated miRNAs and incidentally almost all of them seem to affect wnt signaling for the mediation of their cellular affects. Keeping this consideration in mind, we evaluated wnt signaling as the possible mechanism and evaluated phosphorylation of β-catenin as the marker of wnt signaling. The decision to study this event was because β-catenin is a downstream wnt signaling molecule. Its phosphorylation signals its degradation and therefore an increased phosphorylation of β-catenin represents repressed wnt signaling that often results in reduced metastasis. Reduced phosphorylation of β-catenin results in its translocation to nucleus where its transcriptionally activated many target genes leading to increased metastasis^29^. We establish that c-MYB reduces β-catenin phosphorylation that is suggestive of increased wnt signaling. This process is clearly mediated by miR-21 as suppressing miR-21 abrogates this effect.

In summary, based on the presented evidences, we have provided mechanistic details of cisplatin resistance in ovarian cancer by performing functional studies employing overexpression and silencing of c-MYB and miR-21. We show that c-MYB can profoundly influence cisplatin resistance and, moreover, this involves miR-21 as well as activation of wnt signaling. This needs to be further examined in human patients along with the testing of appropriate therapies for the maximum benefit of ovarian cancer patients.

## Data Availability

All the data is described within the manuscript.
